# Stakeholders’ experiences and perspectives of reproductive genetic carrier screening in gamete donation: a scoping review

**DOI:** 10.1093/humrep/deaf128

**Published:** 2025-07-03

**Authors:** Diya Porwal, Giselle Newton, Julia Mansour, Lisa Dive

**Affiliations:** Graduate School of Health, The University of Technology, Sydney, NSW, Australia; The Centre for Digital Culture and Society, The University of Queensland, Brisbane, QLD, Australia; The Human Genetics Society of Australasia, Hobart, TAS, Australia; Graduate School of Health, The University of Technology, Sydney, NSW, Australia

**Keywords:** reproductive genetic carrier screening, gamete donation, donor conception, assisted reproductive technology, scoping review

## Abstract

**STUDY QUESTION:**

What is known about stakeholders’ experiences and perspectives with reproductive genetic carrier screening (RGCS) in gamete donation?

**SUMMARY ANSWER:**

RGCS has impacted donors’ autonomy, recipients’ decision-making, healthcare professionals’ confidence, and third-party service providers’ management of the donor pool.

**WHAT IS KNOWN ALREADY:**

Growing acceptance of diverse family structures and advances in RGCS technology have driven demand for RGCS in gamete donation, yet its clinical, social, and ethical implications remain poorly understood.

**STUDY DESIGN, SIZE, DURATION:**

A scoping review of four databases (Medline, Embase, CINAHL, and Scopus) with citation searching was conducted to identify original research, position statements, and conference abstracts published in English with an unrestricted date range.

**PARTICIPANTS/MATERIALS, SETTING, METHODS:**

Of the 470 studies identified, 427 were excluded during title and abstract screening and 14 during full-text review based on inclusion and exclusion criteria. For the 29 studies included, data were extracted in excel, and NVivo was used to code data and derive themes.

**MAIN RESULTS AND THE ROLE OF CHANCE:**

Four themes regarding stakeholders’ experiences and perspectives with RGCS in gamete donation were derived: (i) RGCS presented new challenges regarding donor autonomy, informed consent, and result disclosure; (ii) recipients valued RGCS but decision-making was also shaped by cost, time constraints, and genetic literacy; (iii) healthcare professionals supported donors and recipients with RGCS, yet felt unskilled and inexperienced; and (iv) third-party service providers managed donor availability challenges from increased carrier detection through RGCS.

**LIMITATIONS, REASONS FOR CAUTION:**

This review was restricted to articles published in English. A range of terms were used to describe RGCS; thus, it is possible that not all relevant articles were identified in the search. Most included studies were conducted in the USA within a private medical system that permits compensation for gamete donors, which may shape the results and relevance to other countries with differing healthcare systems.

**WIDER IMPLICATIONS OF THE FINDINGS:**

Our findings suggest that the growing demand for RGCS technology may impact donors’ willingness to donate and the availability of donor gametes. Third-party service providers may refine their exclusion criteria to include donors with a positive carrier status in the donor pool or increase the involvement of recipients in donor selection. Healthcare professionals working in gamete donation require more guidance and training on RGCS. Further research is required to establish a more robust evidence base regarding how RGCS impacts stakeholders and to establish clearer guidelines regarding RGCS in gamete donation.

**STUDY FUNDING/COMPETING INTEREST(S):**

This research did not receive any specific grant from funding agencies in the public, commercial, or not-for-profit sectors.

**REGISTRATION NUMBER:**

n/a.

## Introduction

An increasing number of people are using donated gametes to conceive, including across heterosexual and Lesbian, Gay, Bisexual, Trans & Queer+ (LGBTQ+) couples, as well as single intending parents (henceforth recipients) (Sawyer *et al.*, 2013; Salazar *et al.*, 2023). Recipients using donor gametes have specific expectations about the gamete donor’s health, and screening is conducted to gain a detailed medical, genetic, psychosocial, and reproductive history to maximize the health of those conceived via this reproductive intervention (Scheib *et al.*, 2000; Rodino *et al.*, 2011; Nurudeen *et al.*, 2013). There is also growing recognition that donor-conceived people should be able to access detailed medical history and genetic information relating to their donor(s) (Crawshaw *et al.*, 2015).

Reproductive genetic carrier screening (RGCS) is a genetic test that seeks to identify individuals who are carriers of autosomal or X-linked recessive conditions. Information about carrier status can be helpful in preconception planning and decision-making as it indicates the likelihood of having a child with a recessive genetic condition (Rogers *et al.*, 2024). In recent decades, the advent of high-throughput platforms, new sequencing approaches, and projects aimed at sequencing the human genome have reduced financial and technological barriers to RGCS and allowed for the development of broader RGCS approaches that screen for multiple conditions simultaneously (Lazarin and Haque, 2016; Claussnitzer *et al.*, 2020; Payne *et al.*, 2021). One notable domain in which RGCS has been adopted is in the context of donor conception, whereby donors are screened by ART clinics and gamete donor agencies (henceforth third-party service providers) to improve safety and expand service delivery (Pennings, 2020; Hudson *et al.*, 2025). Research has shown that requests for RGCS of gamete donors have increased significantly (Callum and Isley, 2016; Hudson *et al.*, 2025).

Donor conception presents a unique context for RGCS, with implications for multiple stakeholders, including gamete donors themselves, their biological relatives, recipients, donor-conceived individuals, healthcare professionals, and third-party service providers. These stakeholders may have overlapping or conflicting agendas (Daar *et al.*, 2019). For example, although donors undergo RGCS for reproductive purposes, their primary motivation is not typically personal genetic risk assessment, and they are often excluded from reproductive decision-making (Accoe, 2023). Additionally, many country-specific factors can influence stakeholders’ priorities and agency in gamete donation, such as donor compensation (varying from prohibition of commercial inducement to unregulated markets) and identifiability (where some countries have donor-linking while others have so-called ‘anonymous’ donation, albeit in the age of direct-to-consumer genetic testing), as well as distinct funding arrangements for RGCS and pre-implantation diagnosis (Delatycki *et al.*, 2020; Shapiro *et al.*, 2021; Alon *et al.*, 2024; Hudson *et al.*, 2025). Healthcare professionals and third-party providers must balance recipients' reproductive autonomy with obligations to inform donor-conceived individuals of relevant genetic risks and manage the potential implications for donors (Mertes *et al.*, 2018). This can raise ethical and legal questions regarding consent, ownership, and disclosure of results, differing from other RGCS contexts (Hudson *et al.*, 2025).

Given the significant growth in demand for gamete donation and the adoption of RGCS, it is important to consolidate what is known about how RGCS impacts the experiences and perspectives of stakeholders associated with gamete donation. Thus, this study aimed to:

1. Summarize and synthesize the existing research on the experiences and perspectives of various stakeholders with RGCS.
2. Identify gaps in the literature regarding the experiences and perspectives of stakeholders with RGCS and provide future directions for research into the ethical, social, and clinical considerations for RGCS in the context of gamete donation.

## Materials and methods

A scoping review methodology was selected as the most suitable approach to address the exploratory research aims (Peters *et al.*, 2020). The review followed frameworks developed by key authors in the field (Popay *et al.*, 2006; Peters *et al.*, 2020) to respond to the following research question: What are stakeholders’ experiences and perspectives with RGCS in gamete donation?

### Inclusion/exclusion criteria and search strategy

This study’s inclusion and exclusion criteria were developed using the population, concept, and context of the research question (see Table 1) (Peters *et al.*, 2020). Peer-reviewed published studies and abstracts were included, as well as guidelines and position statements, which represent the consensus of groups of experts and organizations. Case studies, reviews, grey literature, and opinion and commentary articles were excluded from analysis, as these sources express individual viewpoints or interpretations. Initial keywords were identified through iterative literature searches based on the research questions and inclusion criteria, and search terms were refined and adapted for different databases with the help of a librarian (Table 2). The literature search was conducted using four electronic databases: Medline, Embase, CINAHL, and Scopus in December 2024 and include articles published in English. The search terms for Medline are outlined in Table 3. The search was not limited by a date range, given that policy and practice have not always kept pace with technological advancements and issues raised in older research may remain relevant today. Search results were uploaded to Covidence and deduplicated. Forward and backward citation searches were conducted on the included papers. Authors of conference abstracts and posters were contacted for pre- or post-print manuscripts, and those provided were included.

**Table 1.** Inclusion and exclusion criteria used for screening articles.

	Included	Excluded
**Population**	Sperm, egg donors Sperm, egg recipients Health professionals Donor-conceived people Staff at gamete banks Staff at IVF clinics Expert organizations	
**Concept**	Experiences and opinions associated with genetic screening of gamete donors Post-screening experiences and psychological outcomes	Experiences and perspectives of various stakeholders towards gamete donation as a whole
**Context**	Empirical studies Position papers Guidelines and recommendations by professional bodies Conference abstracts and posters	Articles published in a language other than English Opinion articles presenting the views of individuals Case studies Books Review articles Unpublished dissertations

**Table 2.** Search strategy used in various databases.

**Population**	Gamete donors/donation Sperm donors/donation Semen donors/donation	Egg donor/donation Oocyte donor/donation Ovum donor/donation
**Concept**	Carrier screening Genetic screening Preconception screening Genetic testing	Carrier testing Preconception testing Genetic counselling
**Context**	Barriers Deterrents Obstacles Challenges Experiences Perspectives	Attitudes Concerns Dilemmas Motivations Expectations Perceptions

**Table 3.** Full search strategy for Medline.

Search	Query
#1	(egg don* OR oocyte don* OR ovum don* OR sperm don* OR semen don* OR gamete don*).mp
#2	Genetic carrier screening/
#3	Genetic testing/
#4	(carrier test* OR carrier screen* OR genetic test* OR genetic screen* OR carrier detection OR donor counsel? ing OR preimplantation test*).mp
#5	#2 OR #3 OR #4
#6	Attitude/
#7	Motivation/
#8	(barrier* OR deter* OR obstacle* OR challeng* OR experienc* OR perspective* OR attitude* OR concern* OR dilemma* OR motivati* OR expect* OR perception*).mp
#9	#6 OR #7 OR #8
#10	#1 AND #5 AND #9

### Data screening, extraction, and analysis

The first 10% of articles were screened by D.P. and G.N. with a high level of agreement (0.81–1.00 Cohen's kappa), and conflicts were resolved through discussion with J.M. and L.D. (McHugh, 2012). The remaining articles were reviewed by D.P. and validated by G.N. An iterative process of data synthesis performed by D.P. involved summarizing the main features of included studies in Excel, namely the year and location of publishing, study methodology, third-party service provider, and key findings, developing a preliminary synthesis exploring the relationships within and between studies, and reviewing theme clusters with G.N. and J.M. (Peters *et al.*, 2020). NVivo (Version 14, Lumivero) was used to code data, group themes by stakeholder category, and identify sub-themes within and between articles by D.P. All authors subsequently contributed to theme consolidation over three discussions and worked on multiple iterations of the manuscript.

## Results

**Figure 1.**
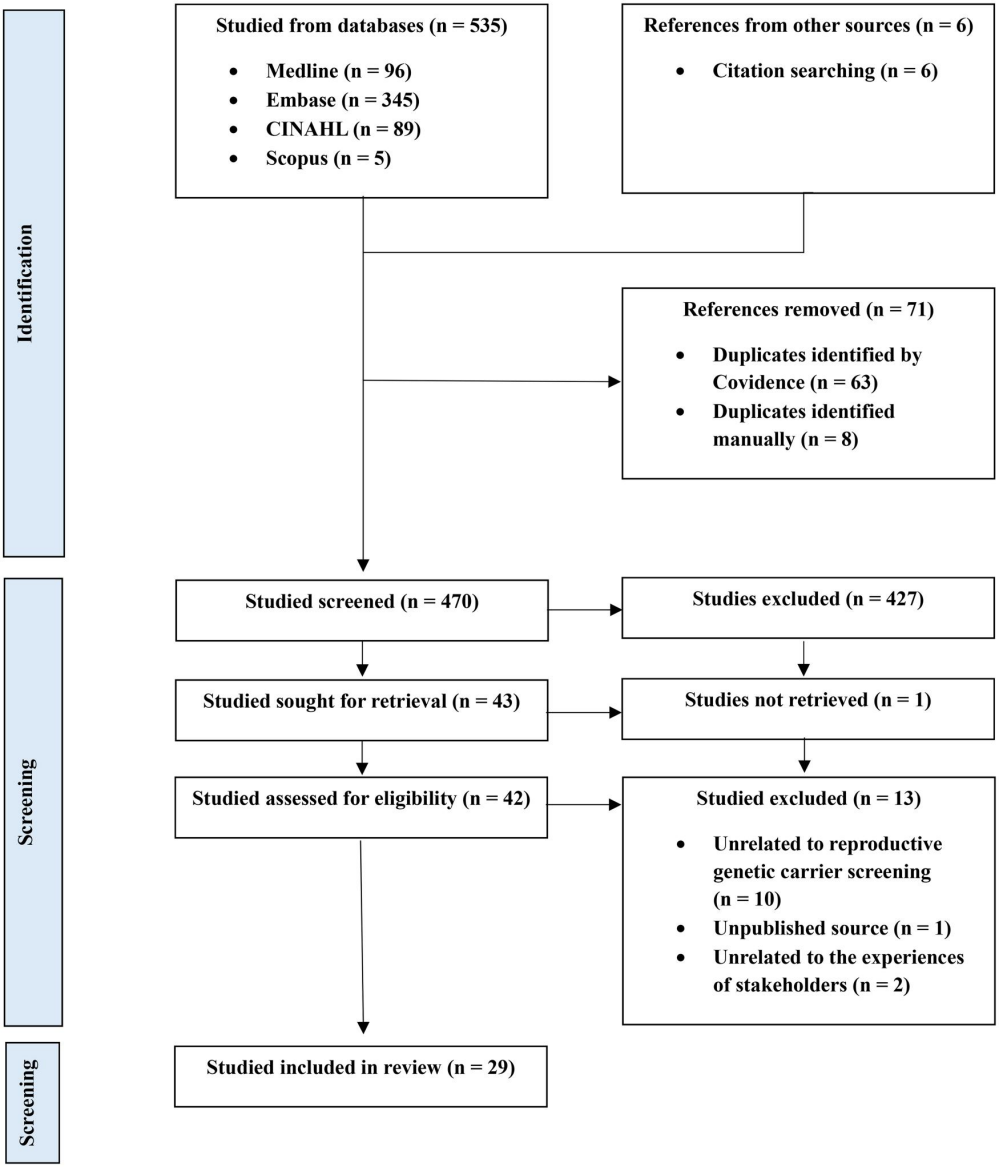
PRISMA flow diagram.

Of the 470 studies identified in the database search, 427 were excluded during screening by title and abstract, and 14 during full-text review (see the PRISMA in Fig. 1). The 29 articles included in this scoping review were published between 2008 and 2024, with most published after 2016 (76%). Many studies published after 2016 were presented as peer-reviewed conference abstracts (64%), and full-text versions were not available for these studies. Most studies were published in the USA (69%), with fewer published in Australia, Belgium, Denmark, Netherlands, and Israel. Most of the studies were quantitative (70%) and used a questionnaire survey methodology. Details of the types of participants and study methodologies are included in Table 4.

**Table 4.** Stakeholders discussed, country of publication, and research methodology for the included articles.

Year	Authors	Stakeholders discussed					Continent/country			Method			
		Donors	Recipients	Donor-conceived people	Health Care Provider	Third-party service providers	USA	EU	Others	Qual	Quant	Position papers/Guidelines	Others
2008	Baker *et al.*		×				×				×		
2010	Sims *et al.*					×	×				×		
2011	Vance				×		×				×		
2011	Lim *et al.*	×	×		×	×	×				×		
2013	Sawyer *et al.*		×					×			×		
2014	Callum *et al.*					×	×				×		
2014	Dondorp *et al.*	×	×					×				×	
2016	Klitzman				×		×				×		
2016	Callum and Isley		×				×				×		
2016	Madsen and Shoul					×		×					×
2017	Jackson *et al.*		×					×			×		
2017	Baldwin				×		×				×		
2018	Homsy *et al.*		×				×				×		
2018	Melnick		×				×				×		
2018	Amor *et al.*	×	×						×	×			
2019	Isley *et al.*	×				×	×				×		
2020	Glenn *et al.*				×		×				×		
2020	Vance				×		×						×
2021	Pennings *et al.*	×					×	×			×		
2021	ASRM and Practice Committee of the American Society for Reproductive Medicine	×	×		×		×					×	
2021	Skytte *et al.*	×					×						
2021	Luque *et al.*	×					×				×		
2022	Park *et al.*					×	×				×		
2022	Aharoni *et al.*		×						×		×		
2022	Baldwin and Callum												
2023	Callum		×				×						×
2023	Vanbelleghem *et al.*		×					×			×		
2024	Klein *et al.*				×			×		×			
2024	Kalscheur *et al.*		×				×			×			
Totals	29	8	14	0	8	6	20	7	2	3	19	2	3

Analysis identified four themes relating to stakeholders' experiences and perspectives on RGCS in gamete donation: (i) donors’ attitudes, engagement, and experiences; (ii) recipients' reproductive choices and plans; (iii) healthcare professionals’ genetics knowledge, skills, and confidence; and (iv) third-party service providers’ management of donor eligibility (see Table 5). No research was identified that explored donor-conceived people’s experiences and perspectives regarding the use of RGCS in donor conception or how individuals have used or would use information obtained through RGCS.

**Table 5.** Themes derived from the summary and synthesis of the existing literature.

Theme	Subtheme
Donors’ attitudes, engagement and implications	Reproductive Genetic Carrier Screening (RGCS) may reveal health and health management information with relevance for donors, and their biological relatives (Dondorp *et al.*, 2014; Luque *et al.*, 2021) Donor attitudes to genetic carrier screening: Donors are comfortable with the existing level of screening (Amor *et al.*, 2018) Willingness to have results shared with recipients (Amor *et al.*, 2018) Positive/uncertain attitude to future screening, expansion of testing (Amor *et al.*, 2018; Pennings *et al.*, 2021) Concerns about impacts for them: possible revelation of medical issues of which they are unaware, impacts long-term psychological health (Amor *et al.*, 2018) Concerns about broader impacts: genetic selection (Amor *et al.*, 2018)
Recipients’ reproductive choices and plans	Recipients considered RGCS results as a key factor in donor selection and the selection of third-party service providers, among other factors (Jackson *et al.*, 2017; Homsy *et al.*, 2018; Melnick, 2018; Aharoni *et al.*, 2022) How RGCS results impacted donor selection depended on: Recipients’ trust in the third-party service provider’s donor screening process (Kalscheur *et al.*, 2024) Recipients’ comprehension of RGCS results and access to genetic counselling (Lim *et al.*, 2011; Melnick, 2018; Vanbelleghem *et al.*, 2023) The availability of substitute donor samples (Amor *et al.*, 2018) The severity of the condition carried by the donor (Jackson *et al.*, 2017) The recipient's age (Vanbelleghem *et al.*, 2023) The duration between scheduled medical insemination and identification of donor’s carrier status (Vanbelleghem *et al.*, 2023) RGCS introduces new barrier for recipients: Requests for RGCS are complicated by the donor’s availability and willingness to participate (Callum and Isley, 2016) Recipients mentioned delay in reproductive plans due to long turnaround times for RGCS (Callum and Isley, 2016; Callum, 2023) Recipients may bear the additional cost of RGCS (Dondorp *et al.*, 2014; Callum and Isley, 2016)
Healthcare professionals’ genetics knowledge, skills, and confidence	Healthcare professionals assessed family history information to determine the appropriate RGCS was undertaken (Vance, 2022) Healthcare professionals provided genetic counselling to recipients regarding the donors' carrier status (Homsy *et al.*, 2018; Vanbelleghem *et al.*, 2023) Healthcare professionals provided genetic counselling to donors about their RGCS results (Glenn *et al.*, 2020) Healthcare professionals felt unskilled and inexperienced with RGCS due to: Challenges with interpretation (Glenn *et al.* 2020) Lack of knowledge (Klein *et al.*, 2024) Complexity of counselling (Baldwin, 2017; Klein *et al.*, 2024) Healthcare professionals indicated they felt ethically conflicted regarding donor engagement relating to: Donors that decline RGCS may not be permitted to participate as donors (Glenn *et al.*, 2020) Appropriate informed consent may not be sought from donors (Lim *et al.*, 2011; Klitzman, 2016) Donors may not be appropriately supported through the process of RGCS (Lim *et al.*, 2011)
Third-party service providers’ management of donor eligibility	RGCS increases the detection of carriers, making it challenging for third-party service providers to exclude donors and maintain the donor pool (Callum and Isley, 2016; Baldwin and Callum, 2022) Third-party service providers are developing new processes to address the issue: providing recipients more autonomy in donor selection and incorporating resources to support them, additional variant analysis (Callum and Isley, 2016; Madsen and Schou, 2016; Baldwin and Callum, 2022; Park *et al.*, 2022; Vanbelleghem *et al.*, 2023)

### Donors’ attitudes, engagement, and experiences

RGCS may reveal information relevant to the health and health management of donors and their biological relatives (Dondorp *et al.*, 2014; Luque *et al.*, 2021). In a retrospective study by Luque *et al.* (2021), clinically significant results were identified in 1 in 50 donors. Furthermore, advances in RGCS technology mean that donor samples may be repeatedly requalified, with new health information being made available at any time after donor qualification (Callum *et al.*, 2014; Isley *et al.*, 2019). Donors' attitudes towards the information that may be obtained through RGCS varied across studies. One study found that donors felt comfortable with RGCS when screening included four genes (Amor *et al.*, 2018). However, when questioned about further screening, donors expressed a willingness to undergo screening and have results shared with recipients but were apprehensive about receiving the results themselves (Amor *et al.*, 2018). Donors raised questions and concerns about the necessity for expanding the scope of RGCS, the long-term psychological consequences of more extensive RGCS results, and genetic selectivity (Amor *et al.*, 2018). In contrast, other studies suggested that a majority of donors have a positive attitude towards RGCS (Pennings *et al.*, 2021; Skytte *et al.*, 2021). A study reporting on the experiences of sperm donors in Denmark and the USA found that 82.5% of donors wanted to understand their full RGCS results, but 10% preferred to limit the results they received. Notably, one in six donors stated that they did not know how they would personally respond if RGCS became more comprehensive (Pennings *et al.*, 2021).

### Recipients' reproductive choices and plans

The extent and results of RGCS may have an impact on recipients’ choice of third-party service provider through which to conceive and/or select a donor. One study found that some participants selected a specific third-party service provider over others because it offered more comprehensive RGCS for donors (Kalscheur *et al.*, 2024). Recipients also described RGCS results as an important factor in donor selection and indicated support for RGCS of donors beyond the recommended guidelines (Sawyer *et al.*, 2013; Jackson *et al.*, 2017; Homsy *et al.*, 2018; Melnick, 2018; Aharoni *et al.*, 2022). One study found that some recipients would be willing to pay for additional screening of donors (Sawyer *et al.*, 2013).

Noting recipients' positive attitudes towards RGCS and their desire for donors to be screened, their actual selection of donor samples was influenced by other factors. Studies have identified that the cost of screening, availability of alternative donor samples, severity of the condition carried by the donor, the recipient's age, the time to the scheduled medical insemination, and recipients’ trust in the third-party service provider’s donor screening process may influence a recipient’s prioritization of RGCS results in donor selection (Baker *et al.*, 2008; Jackson *et al.*, 2017; Amor *et al.*, 2018; Vanbelleghem *et al.*, 2023; Kalscheur *et al.*, 2024). Furthermore, recipients' own experience with genetic screening and genetic counselling influences their selection of donors (Melnick, 2018; Vanbelleghem *et al.*, 2023; Kalscheur *et al.*, 2024). Some recipients described genetic information as difficult to comprehend, overwhelming, and a factor overcomplicating the donor selection decision (Kalscheur *et al.*, 2024). In contrast, recipients who had undergone RGCS themselves were more comfortable incorporating RGCS results during donor selection (Melnick, 2018). They were more likely to order vials from donors who had also undergone RGCS and were more likely to select a donor with a positive carrier status compared to donors who had not undergone RGCS (Melnick, 2018). In one study, 41.3% of recipients proceeded with samples from sperm donors with a positive carrier status following genetic counselling (Vanbelleghem *et al.*, 2023).

Evidence is limited regarding how RGCS impacts timeframes and costs in donor conception. Requests for RGCS are complicated by the donor's availability and willingness to participate (Callum and Isley, 2016). Recipients reported that long turnaround times for RGCS delayed their reproductive plans (Callum and Isley, 2016; Callum, 2023). Furthermore, RGCS may introduce new costs in donor conception. One study found that 24% of recipients would accept any additional RGCS recommended by a healthcare professional. This may increase costs for recipients if they are found to be a carrier of a rare condition, and donor testing is only accessible through a few laboratories (Dondorp *et al.*, 2014; Callum and Isley, 2016).

### Healthcare professionals’ genetics knowledge, skills, and confidence

Professional guidelines and empirical studies recommended that healthcare professionals adequately support donors and recipients through the process of RGCS (Callum *et al.*, 2014; Dondorp *et al.*, 2014; Glenn *et al.*, 2020; ASRM and Practice Committee of the American Society for Reproductive Medicine, 2021; Vanbelleghem *et al.*, 2023). Healthcare professionals assessed family history information to determine the appropriate RGCS to undertake, provided genetic counselling to donors about their RGCS results, and advised recipients on the donors' carrier status (Lim *et al.*, 2011; Vance, 2011; Homsy *et al.*, 2018; Glenn *et al.*, 2020; Baldwin and Callum, 2022; Vance, 2022; Vanbelleghem *et al.*, 2023). Following RGCS results disclosure, recipients also sought advice from healthcare professionals about donor selection (Callum and Isley, 2016; Homsy *et al.*, 2018). Studies indicate that fertility healthcare professionals felt unskilled and inexperienced regarding the RGCS of gamete donors (Glenn *et al.*, 2020; Klein *et al.*, 2024). Most healthcare professionals in one study drew attention to the challenges of interpretation, stating that RGCS should only be offered if the clinical significance of genetic variation is understood (Glenn *et al.*, 2020). Healthcare professionals also identified difficulties in supporting patients engaging with RGCS due to a lack of knowledge and the complexity of counselling (Baldwin, 2017; Klein *et al.*, 2024). Additional challenges may emerge due to variations in technology and interpretation by individual screening laboratories, the requalification of donor samples, and the requirement to update the list of conditions routinely included in the RGCS of prospective donors (Sims *et al.*, 2010).

Healthcare professionals have also indicated they feel ethically conflicted regarding the engagement of gamete donors undertaking RGCS; namely, prospective donors who decline RGCS may not be permitted to participate as donors, appropriate informed consent may not be sought from donors mandated to undergo RGCS, and donors may not be adequately supported through the process of RGCS (Lim *et al.*, 2011; Klitzman, 2016; Glenn *et al.*, 2020). A survey of practices of third-party service providers in the USA found that egg donors were only consulted about the RGCS to be performed on their sample at 28 out of the 44 (63%) clinics participating in this study. Additionally, egg donors were only counselled about the results of their family history assessment at 26 clinics (59%) and about the results of their RGCS results at 40 clinics (90%) (Lim *et al.*, 2011).

### Third-party service providers’ management of donor eligibility

RGCS increases carrier detection, meaning more donors within the donor pool are identified as carriers of a recessive genetic condition (Callum and Isley, 2016; Baldwin and Callum, 2022). This can create challenges for third-party service providers to maintain the size of their donor pool (Callum and Isley, 2016). Some strategies used by third-party service providers to address this challenge included refining their exclusion criteria, involving recipients in donor selection, and incorporating resources to support recipients (Madsen and Schou, 2016; Park *et al.*, 2022; Vanbelleghem *et al.*, 2023). One study described a digital platform where a donor's carrier status regarding registered genetic conditions is made available to potential recipients (Madsen and Schou, 2016). A different study described incorporating genetic counselling for recipients to assist them to interpret genetic risk according to their unique circumstances (Vanbelleghem *et al.*, 2023). Another approach taken was to conduct additional variant analysis on a donor's positive genetic carrier screening result (Park *et al.*, 2022). Of the participants with a positive genetic carrier screening result, 93.9% were allowed to proceed as donors based on the specific variant analysis and the absence of evidence of increased morbidity or mortality (Park *et al.*, 2022).

## Discussion

This is the first scoping review to explore the experiences and perspectives of stakeholders involved in gamete donation regarding the RGCS of gamete donors. Twenty-nine articles were included that investigated the experiences and perspectives of gamete donors, recipients, healthcare professionals, and third-party service providers implicated in the RGCS of gamete donors. No studies were available regarding the experiences or perspectives of donor-conceived people. Results revealed that RGCS has created new considerations and challenges for all stakeholders involved in gamete donation.

Gamete donors indicated mixed views about the current scope of RGCS and shared concerns about engaging in further testing for a broader range of conditions (Amor *et al.*, 2018; Pennings *et al.*, 2021). Inconsistent consent, support, and guidance in navigating RGCS may impact donor willingness to donate and therefore could have consequences for the accessibility of gamete donation (Luque *et al.*, 2021). However, these findings must be interpreted cautiously because only four articles were included in this study regarding the experiences and perspectives of gamete donors with RGCS, and all articles considered the views of existing donors. Furthermore, gamete donors in the most recent studies were more comfortable with RGCS (Pennings *et al.*, 2021; Skytte *et al.*, 2021), potentially suggesting a shift in donors' attitudes and third-party service providers’ execution of RGCS. Further research is required to explore the views of prospective donors, including those who may decline to donate due to RGCS, to better understand the broader impact of adopting, and mandating RGCS for gamete donors.

Our findings draw attention to the ethical complexities regarding ‘ownership’ over a donor’s RGCS results. In accordance with our findings, indicating that donors may not be consulted or supported in the process of RGCS (Lim *et al.*, 2011; Klitzman, 2016; Glenn *et al.*, 2020), one unpublished study from the USA found that where donors are financially compensated, their RGCS results may not be disclosed to them, or appropriate support may not be provided to interpret the significance of the results (Hudson *et al.*, 2025). The impact of compensation on donors' experiences and behaviour regarding RGCS should be further investigated in future research, particularly as countries consider the role of donor compensation in the context of a growing demand for donor gametes (Goedeke *et al.*, 2020; Vanbelleghem *et al.*, 2023).

The potential for new genetic findings to emerge post-donation through requalification introduces ongoing consent considerations and may extend the nature of the relationships between the third-party service provider and donors, recipients, and donor-conceived people beyond the initial donation (Sims *et al.*, 2010; Callum *et al.*, 2014; Baldwin, 2017). While research regarding donor consent in RGCS is sparse, egg donors in a 2021 study indicated that they did not feel well-informed about the potential long-term consequences of egg donation during the consent process (Tober *et al.*, 2021). Further research is required to determine the frequency and nature of consent sought from donors and to develop guidelines regarding recontacting donors, recipients, and donor-conceived people.

Another issue absent from the existing literature is gamete donors' concerns about family communication around RGCS results (although a recent case study by Lemaire *et al.*, (2025) explores the implications of variant identification in a donor-conceived individual on the donor and their relatives). Studies in other genetic screening contexts demonstrate that many patients find it burdensome to inform family members of genetic risks and worry that disclosure will cause them distress (Phillips *et al.*, 2021). As such, potential donors may be dissuaded from participating in gamete donation based on the perceived burden of responsibility of family communication. Additional research should consider family communication regarding genetic screening in the context of donor conception.

Despite multiple influencing factors in donor selection, recipients valued RGCS information, and many were comfortable selecting donors with a positive carrier status (Melnick, 2018; Vanbelleghem *et al.*, 2023), although such donors may be excluded in some jurisdictions (Payne *et al.*, 2021). Factors contributing to selecting a donor with positive carrier status include the recipient’s personal experiences with genetic screening and genetic counselling, a lack of alternative donor samples, the severity of the condition carried by the donor, the recipient's age, the time to the scheduled medical insemination, and the recipient’s trust in third-party services’ screening of donors (Baker *et al.*, 2008; Jackson *et al.*, 2017; Amor *et al.*, 2018; Vanbelleghem *et al.*, 2023; Kalscheur *et al.*, 2024). New strategies by third-party service providers to include donors with a positive carrier status in the donor pool by refining their exclusion criteria and involving recipients in donor selection may indicate a shift in the knowledge and subsequent attitudes towards the application of genetic information.

Prior research involving donor-conceived individuals in other contexts suggests that genetic information plays a meaningful role in shaping identity, such as during discussions around resemblance and family history (Indekeu and Hens, 2019). Our findings highlight that the role of RGCS information within this context remains largely unexplored. Future research should investigate whether, and how, donor-conceived people value and use RGCS results and whether their experiences differ from those spontaneously conceived.

Global professional bodies have emphasized healthcare professionals’ duty to inform patients using donor gametes about RGCS (Capalbo *et al.*, 2022). However, findings from this review indicate that many fertility professionals feel unprepared to guide donors and recipients, especially on RGCS (Glenn *et al.*, 2020; Klein *et al.*, 2024). Studies show they are hindered by the lack of clear recommendations and expert guidance (Henneman *et al.*, 2016). This may have implications for patient care, as a lack of confidence in genetic technology may lead healthcare professionals to neglect patients’ psychosocial needs (Das Gupta *et al.*, 2021). Various countries are promoting genetics-focused professional development and collaboration between genetic and non-genetic healthcare professionals to better support patients with RGCS (Henneman *et al.*, 2016; Cornel, 2019; Douma *et al.*, 2019; Edwards and Laing, 2022).

This study has a number of strengths and some limitations. Consistent with a scoping review methodology, no quality assessment was made of included papers in this review. The heterogeneity of studies, including participant types, continent/countries of origin, and methodologies, limited our capacity to compare results between studies. Given the distinct terms used to describe RGCS, it is possible that not all relevant articles were identified through this review. Most studies were conducted in the USA, within a private medical system that permits compensation for gamete donors, which may shape our results and affect their applicability to other kinds of healthcare systems. Nevertheless, our findings suggest that RGCS introduces new considerations for all stakeholders involved in gamete donation. Thus, this review provides a foundation for exploring the impact of RGCS on gamete donation.

## Conclusion

Since 2008, 29 articles have been published on stakeholders’ experiences and perspectives of RGCS in gamete donation. Findings from this review highlight that RGCS influences (i) donors’ attitudes, engagement, and experiences; (ii) recipients' reproductive choices and plans; (iii) healthcare professionals’ genetics knowledge, skills, and confidence; and (iv) third-party service providers’ management of donor eligibility. Research was not identified exploring donor-conceived people’s experiences and perspectives regarding the use of RGCS in donor conception. RGCS introduces new considerations and challenges for all stakeholders involved in gamete donation. Further research evidence is required to better understand how the increasing adoption and advancements in RGCS technology impact stakeholders to establish clearer guidelines regarding RGCS in gamete donation.

## Data Availability

No new data were generated or analysed in support of this research.
